# Negative pressure wound therapy does not decrease postoperative wound complications in patients undergoing mastectomy and flap fixation

**DOI:** 10.1038/s41598-021-89036-3

**Published:** 2021-05-05

**Authors:** L. De Rooij, S. M. J. van Kuijk, E. R. M. van Haaren, A. Janssen, Y. L. J. Vissers, G. L. Beets, J. van Bastelaar

**Affiliations:** 1 Department of Surgery, Zuyderland Medical Center, Postbus 5500, 6130 MB Sittard, The Netherlands; 2 Department of Clinical Epidemiology and Medical Technology Assessment, Maastricht University Medical Center, Maastricht, The Netherlands; 3 Department of Surgery, Antoni Van Leeuwenhoek Hospital, Netherlands Cancer Institute, Amsterdam, The Netherlands; 4 GROW School for Oncology and Developmental Biology, University of Maastricht, Maastricht, The Netherlands

**Keywords:** Breast cancer, Surgical oncology

## Abstract

Patients and breast cancer surgeons are frequently confronted with wound complications after mastectomy. Negative pressure wound therapy (NPWT) is a promising technique for preventing wound complications after skin closure in elective surgery. However, a clinical study evaluating postoperative complications following the use of NPWT, focusing solely on closed incisions in patients undergoing mastectomy, has yet to be performed. Between June 2019 and February 2020, 50 consecutive patients underwent mastectomy with NPWT during the first seven postoperative days. This group was compared to a cohort of patients taking part in a randomized controlled trial between June 2014 and July 2018. Primary outcome was the rate of postoperative wound complications, i.e. surgical site infections, wound necrosis or wound dehiscence during the first three postoperative months. Secondary outcomes were the number of patients requiring unplanned visits to the hospital and developing clinically significant seroma (CSS). In total, 161 patients were analyzed, of whom 111 patients in the control group (CON) and 50 patients in the NPWT group (NPWT). Twenty-eight percent of the patients in the NPWT group developed postoperative wound complications, compared to 18.9% in the control group (OR = 1.67 (95% CI 0.77–3.63), *p* = 0.199). The number of patients requiring unplanned visits or developing CSS was not statistically significant between the groups. This study suggests that Avelle negative pressure wound therapy in mastectomy wounds does not lead to fewer postoperative wound complications. Additionally, it does not lead to fewer patients requiring unplanned visits or fewer patients developing clinically significant seromas.

Trial registration: ClinicalTrials.gov number, NCT03942575. Date of registration: 08/05/2019.

## Introduction

Patients and breast cancer surgeons are frequently confronted with wound complications after mastectomy, such as surgical site infections, seromas, wound dehiscence and wound necrosis^1–3^. These complications have a diverse etiology and prevention and management therefore require a multifactorial approach. Extensive research over the years has provided insight in how to reduce the rate of surgical site infections and wound healing problems. Preoperative measures that can lower the complication rate focus on optimizing intrinsic patient factors such as treating malnutrition or diabetes, and using prophylactic antibiotics^4^. Postoperative negative pressure wound therapy (NPWT) is a promising technique for preventing and managing wound complications in high risk closed incisions^5^. NPWT isolates the wound and manages exudate, thereby preventing infection and cross-contamination. It furthermore reduces edema, stimulates angiogenesis, promotes contraction of the wound edges and maintains a moist wound healing environment. All these factors should contribute to a lower wound infection rate and to fewer problems in wound healing. Evidence suggests NPWT can accelerate healing times and improve patients’ quality of life^6^.

Several clinical studies have demonstrated the effectiveness of NPWT in the management of chronic and complicated wounds^7–11^. A meta-analysis by Semsarzadeh et al. (2015) on the use of NPWT on closed incisions found a 29.4% reduction in SSI; wound dehiscence rates approximately halved when NPWT was used^12^. These studies included patients with closed incisions from different types of procedures. Breast surgery is notorious for seroma formation and seroma related complications^3^. Despite the theoretical large benefit of reducing seroma and its sequelae by using NPWT in breast cancer surgery, its use in standard breast surgery has not been well studied. Cagney et al. (2020) conducted a systematic review regarding the prophylactic use of NPWT for closed incisions after breast surgery, and mainly included papers on complicated breast wounds, and wounds after breast reconstruction (with or without the use of implants) and after reduction mammoplasty^13–20^. They found significantly fewer surgical site complications, such as SSI, seroma, wound dehiscence and wound necrosis. To the best of our knowledge there is at present no clinical study evaluating the risk of postoperative complications following the use of NPWT focusing on closed incisions in patients undergoing mastectomy. The present study aims to estimate the incidence of postoperative complications following the use of NPWT in non-high risk closed incisions.

## Material and methods

### Study design, setting and patient selection

All patients were included and treated in a large teaching hospital in the Netherlands (Zuyderland Medical Center, Sittard). A prospective cohort of consecutive patients (NPWT) was compared to a control group (CON). The patients in the prospective cohort underwent mastectomy with negative pressure wound therapy (NPWT) and were treated between June 2019 and February 2020. The same inclusion criteria and follow-up moments were used as for the control group. The control group comprised of patients that underwent mastectomy with a conventional wound dressing. These patients were treated between June 2014 and July 2018 and had participated in an earlier study evaluating flap fixation and seroma formation (SAM trial 2020)^21,22^.

All patients were recruited from the surgical breast cancer clinic after evaluation for invasive breast cancer or DCIS. Patients undergoing mastectomy or modified radical mastectomy were eligible for inclusion. Direct breast reconstruction was an exclusion criterion.

All procedures performed in this study involving human participants were in accordance with the 1964 Helsinki declaration and its later amendments. Approval by the institutional ethics research committee (METC Zuyd, Zuyderland Medical Center, the Netherlands. METCZ20190024) was granted and all patients provided written informed consent. This trial was registered at ClinicalTrials.gov Identifier: NCT03942575.

### Study interventions

All patients underwent mastectomy with flap fixation using tissue glue. After performing the mastectomy, skin flaps were secured to the underlying pectoral muscle using ARTISS tissue glue. A low vacuum (Armstrong medical) drain was placed in the mastectomy gutter before closing the wound. The skin edges were sutured in one layer using absorbable monofilament sutures (Moncryl 3.0 or V-loc 30 cm), depending on the surgeon’s preference.

#### Negative pressure wound therapy

In negative pressure wound therapy a closed, sealed system is used to achieve negative pressure. A sterile dressing covers the wound and is sealed with an occlusive drape. A non-sterile vacuum pump connected to a suction tube from the wound dressing provides continuous suction. Nominal negative pressure of 80 mmHg is maintained at the wound surface by the Avelle^®^ NPWT Pump. Wound exudate is managed by the dressing through the absorption and gelling capability of Hydrofiber® Technology and via moisture evaporation at the dressing outer surface.

The wound dressing was applied after skin closure in a sterile environment after which the vacuum pump was connected. The negative pressure wound dressing with pump was in place for 7 days. The wound dressing and pump were removed on the seventh day and wound inspection was performed. Dressings were only changed prematurely in cases with saturated wound dressings or in cases with insufficient sealing of the dressing causing a persistent air leak. If the negative pressure wound therapy was terminated prematurely due to pain, bleeding, skin irritation or any other reason, this was reported in the patient file.

#### Drain policy

In all patients, one low suction drain was placed before flap fixation and skin closure. The drain was positioned in the mastectomy gutter, lateral to the pectoral muscle and was connected to a low suction drain bottle (Armstrong medical). Drain output was recorded daily. In patients undergoing mastectomy with axillary clearance the drain was removed when drain volume was < 50 ml/24 h, or after a maximum of five days. In patients undergoing mastectomy without axillary clearance the drain was removed when drain volume was < 50 ml/24 h, or after a maximum of 48 h.

### Follow-up

Patients were evaluated in the outpatient clinic at one week, six weeks and three months postoperatively. The wound dressing and pump were removed after seven days in the outpatient clinic. During the follow-up visits, primary and secondary outcomes were assessed. At any moment during the trial patients could decide to end participation in the study.

### Primary outcome

The number of patients with postoperative wound complications during the first three postoperative months was the primary outcome. The postoperative wound complications were defined as follows:

- Surgical site infection (SSI). Defined as redness, pain, heat or swelling at the site of the incision or by the drainage of pus. Infection rate was measured by (A) the need for antibiotics, (B) seroma aspiration due to infection or C) surgical drainage.
- Wound necrosis. Defined as a wound with necrotic tissue that consists of an accumulation of dead cells, tissue and cellular debris and often requires debridement. Wound necrosis rate was measured by the need for surgical debridement.
- Wound dehiscence or breakdown defined as a wound that ruptures along a surgical incision and the edges of the wound no longer align. Wound dehiscence rate was measured by the need for secondary surgical wound closure and/or the need for vacuum assisted closure (VAC).

### Secondary outcomes

#### Unplanned visits

If patients required unplanned visits to the emergency room or breast cancer clinic during the first three postoperative months, this was documented. Planned visits are standard follow-up outpatient clinic visits which were defined preoperatively. Unplanned visits were defined as any visit to the outpatient clinic which was necessary due to an adverse event related to the mastectomy.

#### Clinically significant seroma

The number of patients with clinically significant seroma (CSS). CSS is defined as:

- Seroma causing delayed wound healing and leading to wound break down, leakage of lymph fluid and/or skin flap necrosis, requiring seroma aspiration or surgical intervention.
- Pain or discomfort caused by large amounts of seroma, characterized by tenseness of the skin, requiring seroma aspiration or surgical intervention.
- Contaminated or infected seroma warranting seroma aspiration or surgical intervention to treat the infection. All patients that underwent seroma aspiration due to infection were also treated with antibiotics for one week (amoxicillin/clavulanic acid 500/125 mg three times daily).

### Sample size calculation

The effect of negative pressure wound therapy has been evaluated in various wound types. However, no studies to date have analyzed NPWT on closed incision wounds after mastectomy. The sample size of 50 was chosen for this study for pragmatic reasons as this number could easily be recruited within a reasonable time frame. Preceding these 50 study patients, 10 patients were included and treated conform the study protocol in a trial phase. These patients are not included in the present study, to avoid a learning curve effect. All eligible control patients were included.

### Statistical analysis

All analyses were performed using SPSS (IBM SPSS statistics for Windows, Version 25).

Baseline characteristics of the prospective and control cohorts were described in detail. Continuous variables were reported as means and standard deviations. Categorical variables were reported as counts and percentages. Differences in baseline characteristics between both cohorts were tested using the independent-samples t-test for continuous variables, and Pearson’s chi-squared test for categorical variables. In case of expected cell counts of less than 5, Fisher’s Exact test was used instead.

The proportion of patients who experienced a postoperative wound complication, who required unplanned visits to the ER or breast cancer clinic, and who developed clinically significant seroma were separately compared between the two groups using multivariable logistic regression analysis, corrected for the potential confounders. Mean total drain output volume was compared between groups using independent samples *t* test.

### Ethical approval

All procedures performed in this study involving human participants were in accordance with the ethical standards of the institutional research committee (ethics committee of Zuyderland Medical Center, Netherlands) and with the 1964 Helsinki declaration and its later amendments.

### Consent to participate

Informed consent was obtained from all individual participants included in the study.

### Consent for publication

The authors affirm that human research participants provided informed consent for publication of the data.

## Results

A total of 161 patients were included in this study. In the retrospective cohort, 111 patients (68.9%) that had been treated between July 2014 and July 2018 were included. Fifty patients (31.1%) were included prospectively and were treated between June 2019 and February 2020 and received the negative pressure wound dressing. Three patients in the prospective cohort were lost to follow-up at the 3-month follow-up visit. One patient moved abroad during follow-up and two patients withdrew from follow-up with no specific reason.

In all patients in the NPWT group the wound dressing was removed on day seven post-operatively. In four patients (8.0%), the wound dressing was prematurely replaced due to air leakage and inadequate suction of the pump. After the dressing was changed, adequate sealing and negative pressure was ensured; the seven-day treatment was completed in all patients. No statistically significant differences between the groups were found in the patient characteristics (Table 1).

**Table 1:** Patient and baseline characteristics.

		Control group *n* = 111	NPWT *n* = 50	*p * value
Age (years)		65.1 ± 13.6	65.4 ± 13.4	0.893
Charlson comorbidity index		5.0 ± 2.2	4.6 ± 1.8	0.297
BMI (kg/m^2^)		27.6 ± 4.9	27.0 ± 5.8	0.47
Number of patients with anticoagulation		26 (23.4)	9 (18.0)	0.44
Number of patients smoking		15 (13.5)	5 (10.0)	0.507
Neoadjuvant chemotherapy		21 (18.9)	15 (30.0)	0.118
Procedure				0.736
Mastectomy ± SN		77 (69.4)	36 (72.0)	
Mastectomy + ALND		34 (30.6)	14 (28.0)	
cT	0	0	3 (6.0)	0.099
	x	3 (2.7)	0	
	Is	8 (7.2)	3 (6.0)	
	1–2	87 (78.4)	36 (72.0)	
	3–4	13 (11.7)	8 (16.0)	
cN	0	80 (72.1)	36 (72.0)	0.701
	1	27 (24.3)	13 (26.0)	
	2	3 (2.7)	0	
	3	1 (0.9)	1 (2.0)	

Continuous variables are presented as means ± SD. Categorical variables in absolute numbers (%).

SN = sentinel node. cT0 = benign tumors/prophylactic. Is = in situ.

### Primary outcome

Fourteen patients (28.0%) in the NPWT group compared to 21 patients (18.9%) in the control group developed postoperative wound complications (odds ratio [OR] = 1.67 (95% Confidence Interval [CI] 0.77–3.63, *p* = 0.199) (Table 2). The majority of these wound complications involved surgical site infections (CON 18.0% vs. NPWT 26.0%). One patient in the control group developed wound necrosis that required surgical debridement. Ten percent of patients in the NPWT group, compared to 3.6% of patients in the control group developed wound dehiscence requiring wound treatment with VAC (OR = 2.97 (95% CI 0.76–11.58), *p* = 0.116) (Table 2).

**Table 2:** Primary outcome: number of patients with postoperative wound complications requiring intervention (%).

	Control group *n* = 111	NPWT *n* = 50	Odds ratio	*p * value
**Postoperative wound complications**	21 (18.9)	14 (28.0)	1.67 (0.77–3.63)	0.199
Surgical Site Infection	20 (18.0)	13 (26.0)	1.60 (0.72–3.54)	0.248
Wound necrosis^*a*^	1 (0.9)	0	0	0.998
Wound dehiscence^*a*^	4 (3.6)	5 (10.0)	2.97 (0.76–11.58)	0.116

^a^Wound necrosis or dehiscence requiring surgical intervention.

### Secondary outcomes

#### Unplanned visits

The number of patients that required additional outpatient department visits was not statistically significantly different between the groups (CON: 36.9% vs. NPWT: 42.0%) (Table 3). The range for unplanned visits was between one and five visits in the NPWT group and between one and 11 in the control group.

**Table 3:** Secondary outcomes: number of patients requiring unplanned visits and developing CSS (%).

	Control group *n* = 111	NPWT *n* = 50	Odds ratio	*p* value
**Unplanned visits**	41 (36.9)	21 (42.0)	1.19 (0.60–2.35)	0.617
**Clinically significant seroma**	15 (13.5)	12 (24.0)	2.02 (0.87–4.71)	0.103
1) Seroma aspiration	13 (11.7)	11 (22.0)	2.13 (0.88–5.15)	0.095
2) Seroma related reoperation or VAC	7 (6.3)	5 (10.0)	1.65 (0.50–5.48)	0.413

#### Clinically significant seroma

Twenty-four percent of the patients in the NPWT group had clinically significant seromas, compared to 13.5% in the control group (OR = 2.02 (95% CI 0.87–4.71), *p* = 0.103 (Table 3)). Although not statistically significant, more patients required seroma aspiration (CON: 11.7% vs. NPWT: 22.0%) and required seroma related reoperation or wound treatment with VAC (CON: 6.3% vs. NPWT: 10.0%). There was a statistically significant difference between the mean total drain output volumes of both groups in favor of the NPWT group. The mean total drain output volume in the control group was 241 ml (± 253) compared to 154 ml (± 202) in the NPWT group (*p* = 0.029).

Correction for potential confounders i.e. axillary clearance, neoadjuvant chemotherapy, the use of anticoagulants, smoking, charlson comorbidity index, BMI and age with multivariable logistic regression, did not result in any statistically significant differences between groups (Table 4).

**Table 4:** Odds ratio adjusted for potential confounders.

	Adjusted odds ratio	*p* value
Postoperative wound complications	2.01 (0.86–4.68)	0.106
Unplanned visits	1.23 (0.60–2.52)	0.577
Clinically significant seroma	2.01 (0.81–5.03)	0.134

Corrected for the potential confounders axillary clearance, neoadjuvant chemotherapy, the use of anticoagulants, smoking, CCI, BMI and age.

## Discussion

This study found that negative pressure wound therapy in patients undergoing mastectomy with skin flap fixation does not lead to a reduction of postoperative wound complications. Although the difference is not statistically significant, there is even an increased wound complication rate from 18.9% in the conventional group versus 28.0% in the negative pressure group. There was no statistically significant difference between the groups in the proportion of patients requiring unplanned visits, nor in the proportion of patients developing clinically significant seroma.

The majority of the postoperative wound complications were surgical site infections, with an incidence of 18% and 26% in the two groups. This is relatively high when compared to the SSI rate found in other studies using closed-incision negative pressure therapy (5–10%)^6,12^. These studies were however performed in surgical wounds other than after mastectomy. Mastectomy wounds are prone to seroma formation, and thus generally a higher rate of surgical site infections^1–3^. The other wound complications were uncommon in the present study. Only one patient required surgical debridement for skin necrosis and nine patients required vacuum assisted closure for a wound dehiscence. No beneficial effect of NPWT was found regarding wound necrosis and wound dehiscence.

Next to seroma formation, the site of the incision can form an extra challenge in negative pressure wound therapy in mastectomy wounds. The incision often ends high in the axillary area and frequently has a curved shape. With the axillary area being a very mobile area and with the rectangular wound dressings, the aforementioned characteristics of the incision can make it challenging to ensure a sealed vacuum during seven days.

During NPWT, the mastectomy wound surface is under external nominal negative pressure of 80 mmHg by the NPWT pump during seven days, possibly having a favorable effect on seroma formation. Clinically significant seroma was therefore one of the secondary outcomes in the study. Contrary to expectations, there was a higher proportion of patients with clinically significant seroma in the NPWT group than in the control group, 24% versus 14%. This difference was not statistically significant, but a difference of this magnitude would be clinically relevant. This finding is at odds with the lower mean total drain output in the NPWT group. This could be a chance finding, or the beneficial effect of the negative pressure on seroma formation during the first seven days could become lost after removal of NPWT.

Regarding the used definition of CSS in this study, it could be argued that seroma aspiration is less invasive than reoperation or wound treatment with VAC and should possibly not be weighed equally. Comparison of the proportion of patients undergoing aspiration and reoperation or wound treatment with VAC between the groups, showed no statistically significant difference.

One of the limitations is that this study is possibly underpowered to detect statistically significant differences, e.g. a 10% difference in CSS. An additional limitation is the non-randomized nature of this study. Changes in daily practice evolving over a period of years, for example a lower threshold for starting wound treatment with VAC, could cause a certain bias in the results. The application of negative pressure wound therapy for only 7 days might negatively influence these study results. It is uncertain whether extending the application of negative pressure wound therapy in mastectomy patients might yield better results. Based on the aforementioned limitations, no conclusions with an impact on standard practice can be drawn from this study. Lastly, it should be mentioned that the conclusions in this study can only be drawn related to application of Avelle negative pressure wound therapy. It is uncertain if application of different negative pressure wound therapy systems, will lead to similar results.

## Conclusion

This is the first study to evaluate postoperative wound complications following the use of negative pressure wound therapy in patients undergoing mastectomy. The results in this study suggest that Avelle negative pressure wound therapy in mastectomy wounds does not lead to fewer postoperative wound complications. Additionally, it does not lead to fewer patients requiring unplanned visits or to fewer patients with clinically significant seroma.

## Data Availability

The datasets during and/or analyzed during the current study are available from the corresponding author on reasonable request.
